# Necrotizing retinitis of multifactorial etiology

**DOI:** 10.22336/rjo.2017.9

**Published:** 2017

**Authors:** Ruxandra Angela Pirvulescu, Cherecheanu Alina Popa, Mihaela Oana Romanitan, Dana Obretin, Raluca Iancu, Danut Vasile

**Affiliations:** * “Carol Davila” University of Medicine and Pharmacy, Bucharest, Romania; ** Ophthalmology Clinic, University Emergency Hospital, Bucharest, Romania; *** Department for Clinical Research, Karolinska Institute, Sodersjukhuset, Stockholm, Sweden; **** Infectious Diseases Department, “Dr. Victor Babes” Clinical Hospital “, Bucharest, Romania; ***** Surgery 1 Clinic, University Emergency Hospital, Bucharest, Romania

**Keywords:** acute necrotizing retinitis, panuveitis, vasculitis, Cytomegalovirus, Herpes Simplex Type 1, corticotherapy

## Abstract

**Introduction.** We present the case of a 73-year-old woman with osteoporosis, who presented to the emergency room with a sudden vision loss and ocular pain in the right eye, which appeared two days before. The patient mentioned loss of appetite, weight loss for three months and low fever for two weeks.

**Materials and methods.** Among the ophthalmological findings, the most important were panuveitis, and large confluent necrotic areas in the peripheral retina. The patient was diagnosed with RE Panuveitis and acute necrotizing retinitis.

**Results.** Blood exams showed leukocytosis and monocytosis, thrombocytosis and anemia. Further investigations showed high levels of Cytomegalovirus (CMV) anti IgG and Herpes Simplex (HS) type 1 virus anti IgM, urinary infection, and secondary hepatic cytolysis. The CT and MRI of the thorax and abdomen showed no sign of neoplastic disease, and no explanation for the CMV infection was found.

The patient received general corticotherapy and antiviral therapy, and, after one month, RE BCVA was 20/ 30.

**Particularity of the case.** Acute necrotizing retinitis in an old patient with CMV and HSV type 1, associated with secondary hepatic cytolysis, without any other immunosuppressive disease and very good outcome.

## Introduction

We present the case of a 73-year-old woman with osteoporosis, who presented to the emergency room with sudden vision loss and ocular pain in the right eye, two days before. The patient mentioned loss of appetite and weight loss for three months and low fever for two weeks. The personal medical history of the patient showed no significant general or ocular pathology.

Clinical eye exam showed:

- RE BCVA = c.f. at 50 cm;

- LE BCVA = 20/ 20;

- RE IOP = 16 mmHg;

- LE IOP = 15 mmHg;

The Goldmann visual field in right eye showed central scotoma on 20 degrees.

Slit lamp examination of the right eye revealed perikeratic injection, posterior synechiae at 180 degrees inferiorly which deformed the pupil, endothelial keratic precipitates in a triangular pattern, flare in the anterior chamber and Tyndall ++. 

**Fig. 1:**
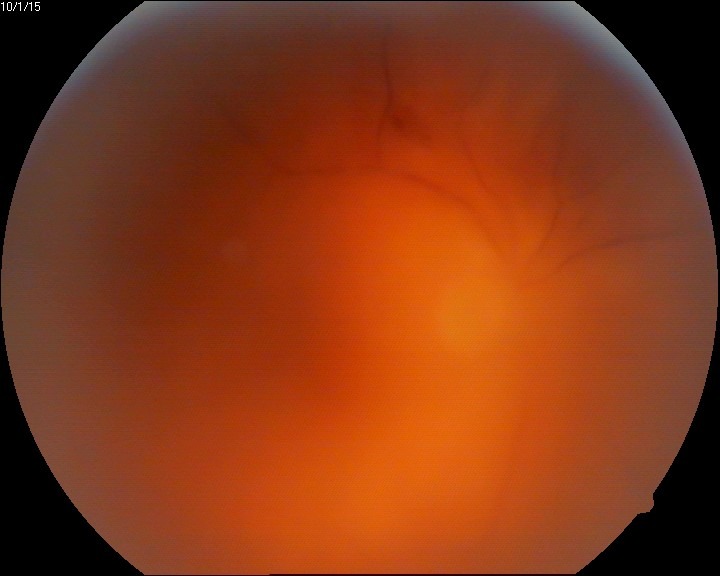
Eye fundus (RE) - vitritis

**Fig. 2:**
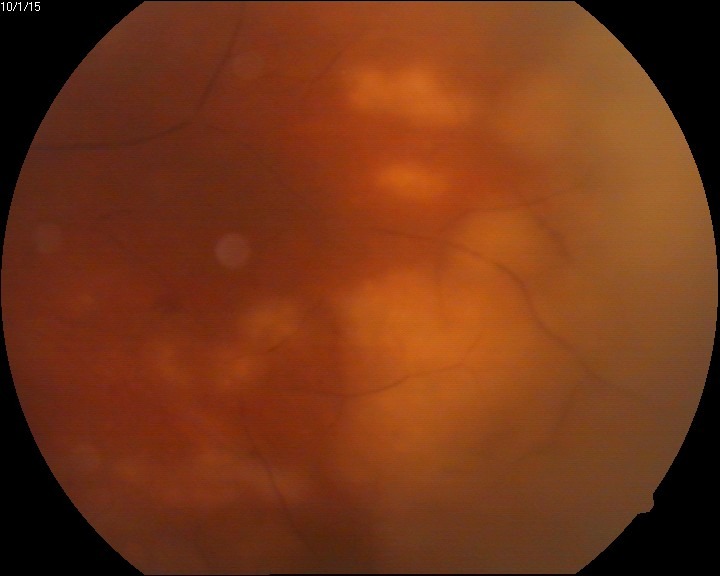
Eye fundus (RE) – large confluent areas of retinal necrosis, narrowed vessels

**Fig. 3:**
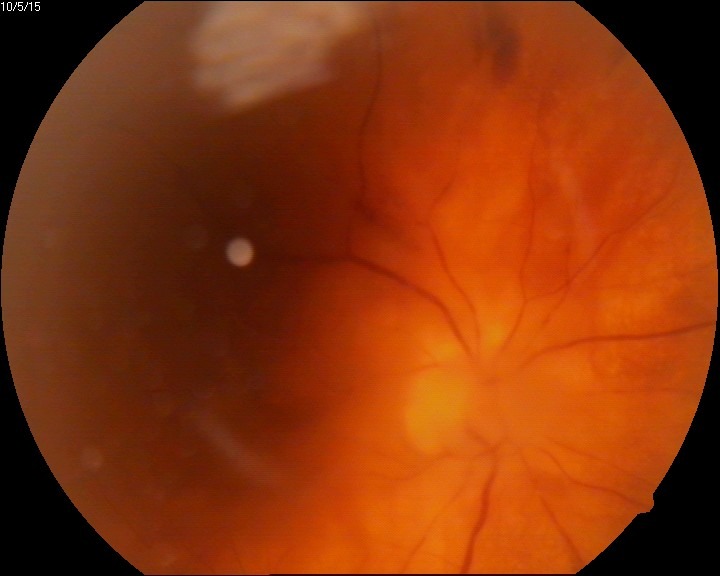
RE - narrowed vessels, peripheral retinal hemorrhage

**Fig. 4:**
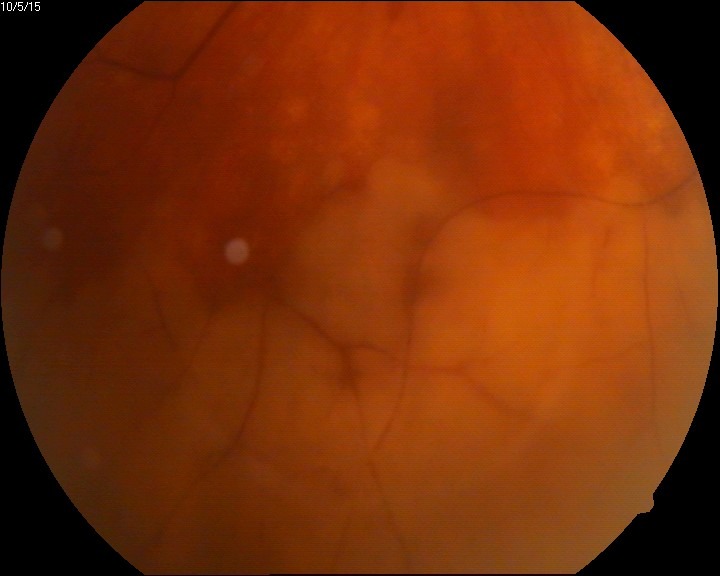
Peripheral retinal necrosis (RE)

**Fig. 5:**
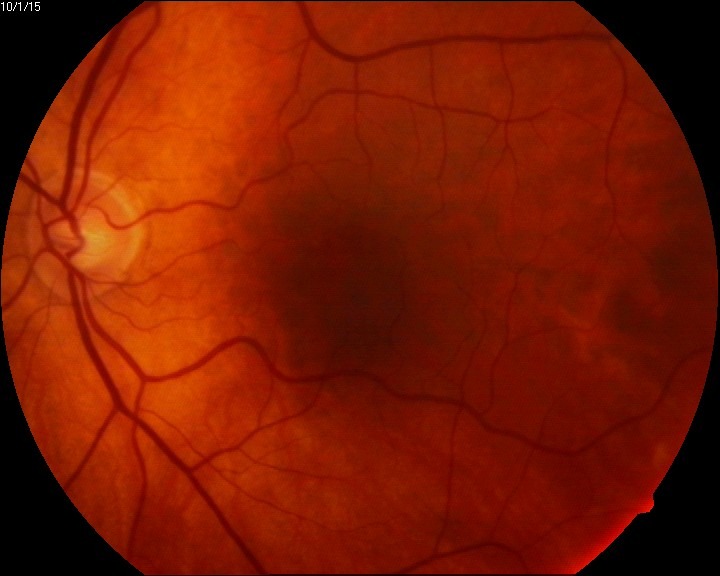
LE Fundus - within normal limits

The eye fundus showed vitreal floaters and vitritis (**Fig. 1**), slightly blurred margins of the optic nerve (unclear due to the inflammation or the vitreal flare), macula with absent foveal reflex, very narrow blood vessels, phantom vessels especially in peripheral retina, perivascular cuffing (vasculitic aspect) (**Fig. 2**), large cotton-wool spots and rare hemorrhages on the retinal periphery (**Fig. 3**), and, most important, large confluent necrotic areas in the periphery and mid-periphery of the retina (**Fig. 2**,**4**). The left eye had a normal aspect (**Fig. 5**).

We established the diagnosis: RE Panuveitis; Necrotizing retinitis

The blood exam revealed:

- LEUCOCITOSIS 11800/ µl (normal range 4000 – 10000/ µl)

- NEUTROPHILIA 10300/ µl (normal range 2000-7000/ µl)

- LYMPHOCYTES 800/ µl (normal range 1000-4000/ µl)

- THROMBOCITOSIS 683000/ µl (normal range 100000-400000/ µl)

- VSH 68 mm/ h (normal range 1-30 mm/ h)

- PCR 3.98 mg/ dl (normal range <0.5 mg/ dl)

- TGO 44 U/ L (normal range 0-32 U/ L)

- TGP 79 U/ L (normal range 0-65 U/ L)

Given the results of the blood test, the patient was referred to hematology and infectious diseases department. Further exams showed:

- AND-CMV 4554 copies/ mL (quantitative test) – latent infection (<200 copies/ mL)

- HSV 1 Ig M 1.8 (positive >1)

- Uroculture - urinary infection (E. Coli)

- Abdominal ultrasound – hepatomegaly, most likely due to secondary hepatic cytolysis

- CT and MRI of the thorax and abdomen showed no sign of neoplastic disease.

The final diagnosis set was RE Necrotizing retinitis.

## Discussion

Acute necrotizing retinitis (ARN) and Progressive Outer Retinal Necrosis (PORN) represent a spectrum of rapidly progressing necrotizing herpetic retinopathies. ARN usually strikes in immunocompetent hosts and continues with vasculitis, iridocyclitis, and vitritis. On the other hand, PORN occurs in immunocompromised persons due to HIV infection or other immunosuppressive conditions. These patients develop a necrotizing retinitis that may rapidly involve the macula as well as the peripheral retina, without significant intraocular inflammation or vasculopathy. The outcomes in both these entities can be devastating and include blindness from complicated retinal detachment and optic atrophy [**1**,**2**]. 

Clinical eye fundus aspect of necrotizing retinitis includes:

- Vitritis (that can be severe);

- Disk edema and retrobulbar optic nerve disease are not uncommon early in the course of ARN;

- Single/ multiple areas of retinal necrosis with distinct borders; 

- Necrotic foci in peripheral retina;

- Extension/ coalescence of foci of retina; necrosis in a circumferential fashion;

- Occlusive vasculopathy with arteriolar involvement (retinal vasculitis is common, usually, primarily we could have arteritis);

- Prominent anterior chamber and vitreous inflammation; 

- Characteristics that support but are not required for the diagnosis: 

• optic neuropathy or atrophy, scleritis, 

• ocular pain 

- Inflammation in the anterior and posterior segments [**1**,**3**];

- Anterior granulomatous or non granulomatous uveitis with keratic precipitates;

- ARN may also present with diffuse scleritis; 

- Therefore, it is imperative to perform a dilated fundoscopic examination of every patient with scleritis [**2**,**3**].

A differential diagnosis is made with several infectious and noninfectious entities, most of these conditions (with the exception of Behcet disease, atypical toxoplasmosis, and bacterial endophthalmitis) progressing at a much slower pace than ARN. 

The retinitis of Behcet’s disease may be indistinguishable from ARN. However, Behcet’s disease is most common in patients of Japanese, Middle-Eastern, or Mediterranean origin, history of oral aphthous ulcers, genital ulcers, or skin lesions (HLA B-51, CD4+, CD8+) [**1**,**3**].

The management of necrotizing retinitis refers, first, to treating the cause of the retinitis. Because most cases of ARN are thought to be caused by Varicella Zoster Virus and HSV, the standard therapy is usually with intravenous acyclovir for 10 to 14 days, followed by a maintenance therapy with oral acyclovir, famciclovir, or valacyclovir. However, more recent data support the induction with oral therapy, for example valacyclovir 1 g three times a day (Aizman, Aslanides). The maintenance therapy for ARN is usually employed for 3 months, in order to reduce the risk of the disease in the fellow eye. It may be used longer in the setting of immunosuppression or multiple recurrences [**4**].

After the first 24 to 48 hours of antiviral therapy, systemic corticosteroids may be introduced to minimize vitritis and the development of vitreous bands, which may contribute to the development of tractional retinal detachment [**3**].

Since the viral involvement was revealed the next day, we considered our patient’s situation as severe, so we decided to administer corticotherapy until the results of the tests came out. The patient received general corticotherapy (Solumedrol 1g/ d for 3 days, then Medrol 0.8mg/ kg in a decreased dosage), and antibiotherapy (Ciprofloxacin 200 mg/ 8h). Antiviral therapy was set the next day, as we had the blood exam final results (Acyclovir 2g/ d for 2 months) [**3**,**4**].

Complications of ARN may be devastating. Usually, the eye is frequently left with 360° of peripheral retinal atrophy, with multiple posterior retinal breaks secondary to retinal necrosis. A combination of rhegmatogenous and tractional retinal detachment may develop secondary to retinal breaks. Optic atrophy frequently develops in patients who suffered from disc edema earlier in the disease [**4**,**5**].

**Fig. 6:**
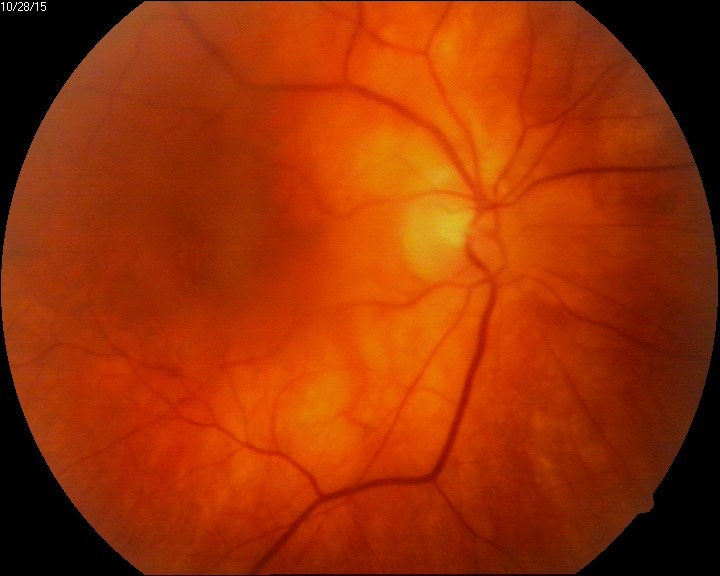
Eye fundus aspect of the RE

The evolution of our patient was quite good, considering the severity of the condition. After one month, RE BCVA was 20/ 30, vitritis disappeared, the vascular aspect normalized and the necrotic areas vanished (**Fig. 6**). 

**Fig. 7:**
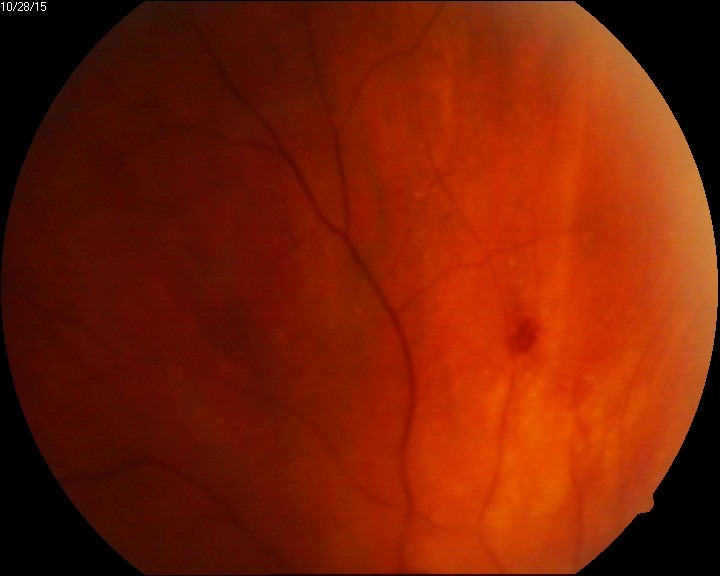
RE eye fundus peripheral aspect

**Conclusion and particularity of the case**

The early diagnosis and treatment of necrotizing retinitis remains the key to a successful management while the prognosis for patients with severe immune dysfunction remains guarded.

The prognosis of untreated ARN has traditionally been poor, with two-thirds of eyes having a visual acuity of 20/ 200 or worse due to retinal detachment, optic atrophy, or retinal pathology [**3**,**4**]. While there are reports of aggressive intervention resulting in better outcomes, overall the prognosis for patients with ARN remains guarded.

The particularity of our case is acute necrotic retinitis in an immunosuppressed old patient, with a strong panuveitis component associated with CMV, HSV type 1 and secondary hepatic cytolysis, without any other associated immunosuppressive disease and any other obvious cause of immunodepression, and with a good outcome. Patient is still under the supervision of the infectious disease department.
